# qPCR Analysis Reveals Association of Differential Expression of *SRR*, *NFKB1*, and *PDE4B* Genes With Type 2 Diabetes Mellitus

**DOI:** 10.3389/fendo.2021.774696

**Published:** 2022-01-03

**Authors:** Waseem Raza, Jinlei Guo, Muhammad Imran Qadir, Baogang Bai, Syed Aun Muhammad

**Affiliations:** ^1^ Institute of Molecular Biology and Biotechnology, Bahauddin Zakariya University, Multan, Pakistan; ^2^ School of Medical Engineering, Sanquan College of Xinxiang Medical University, Xinxiang, China; ^3^ School of Information and Technology, Wenzhou Business College, Wenzhou, China; ^4^ Engineering Research Center of Intelligent Medicine, Wenzhou, China; ^5^ The 1st School of Medical, School of Information and Engineering, The 1st Affiliated Hospital of Wenzhou Medical University, Wenzhou, China

**Keywords:** cDNA datasets, T2DM, differential expressed genes, enrichment analysis, qPCR, expression profiling

## Abstract

**Background:**

Type 2 diabetes mellitus (T2DM) is a heterogeneous, metabolic, and chronic condition affecting vast numbers of the world’s population. The related variables and T2DM associations have not been fully understood due to their diverse nature. However, functional genomics can facilitate understanding of the disease. This information will be useful in drug design, advanced diagnostic, and prognostic markers.

**Aim:**

To understand the genetic causes of T2DM, this study was designed to identify the differentially expressed genes (DEGs) of the disease.

**Methods:**

We investigated 20 publicly available disease-specific cDNA datasets from Gene Expression Omnibus (GEO) containing several attributes including gene symbols and clone identifiers, GenBank accession numbers, and phenotypic feature coordinates. We analyzed an integrated system-level framework involving Gene Ontology (GO), protein motifs and co-expression analysis, pathway enrichment, and transcriptional factors to reveal the biological information of genes. A co-expression network was studied to highlight the genes that showed a coordinated expression pattern across a group of samples. The DEGs were validated by quantitative PCR (qPCR) to analyze the expression levels of case and control samples (50 each) using glyceraldehyde 3-phosphate dehydrogenase (*GAPDH*) as the reference gene.

**Results:**

From the list of 50 DEGs, we ranked three T2DM-related genes (*p* < 0.05): *SRR*, *NFKB1*, and *PDE4B*. The enriched terms revealed a significant functional role in amino acid metabolism, signal transduction, transmembrane and intracellular transport, and other vital biological functions. *DMBX1*, *TAL1*, *ZFP161*, *NFIC* (66.7%), and *NR1H4* (33.3%) are transcriptional factors associated with the regulatory mechanism. We found substantial enrichment of insulin signaling and other T2DM-related pathways, such as valine, leucine and isoleucine biosynthesis, serine and threonine metabolism, adipocytokine signaling pathway, P13K/Akt pathway, and Hedgehog signaling pathway. The expression profiles of these DEGs verified by qPCR showed a substantial level of twofold change (FC) expression $\left(2^{{-\Delta \Delta \text{C}}_{\text{T}}}\right)$ in the genes *SRR* (FC ≤ 0.12), *NFKB1* (FC ≤ 1.09), and *PDE4B* (FC ≤ 0.9) compared to controls (FC ≥ 1.6). The downregulated expression of these genes is associated with pathophysiological development and metabolic disorders.

**Conclusion:**

This study would help to modulate the therapeutic strategies for T2DM and could speed up drug discovery outcomes.

## Introduction

Type 2 diabetes mellitus (T2DM) is a metabolic and complicated condition characterized by insulin resistance and insulin deficiency due to hyperglycemia (1). Currently, about 285 million individuals are estimated to have type 1 and 2 diabetes, which makes up around 90% of the total (2). Tissues including the pancreas, liver, skeletal muscle, adipose, and intestinal tissues have a role in its progress (3), and despite this, several critical variables such as lifestyle, food, obesity, and genetic factors were identified in insulin and T2DM development (4–6). The fundamental mechanisms are still unclear, and due to its high morbidity and increased clinical impact, this disease has become an extremely severe global issue (2). Due to the multifaceted mechanisms and diverse contributory factors of T2DM, there is an urgent need to identify vital biological molecules as potential therapeutic targets and improve the treatment strategies. Different oral and injectable treatments for T2DM are available; however, the principal difficulties associated with the use of these medications are due to a lack of effectiveness, hypoglycemia, weight gain, and increased susceptibility to infections. It is thus a responsibility of the scientist to study the causes and the mechanisms of the disease to properly manage the risks.

Recent developments have shown that diabetes is diverse, with complicated genetic causes. Many studies have demonstrated the significance of various genes in the etiology of disease. Most genes participating in T2DM have been recognized as prospective therapeutic targets; nevertheless, the limited knowledge of the complexity and interaction of these systems has been a significant problem in the development of effective T2DM treatments. Most cases of diabetes include several genes, each of which contributes to an increased risk of type 2 diabetes (2), and similar genes related to T2DM poorly show recognized pathways of insulin signaling (5). The present techniques for finding statistically significant functional classes of genes associated with T2DM have detected regulatory cell cycles (7, 8). However, in T2DM and in the molecular biology of insulin resistance, the functional categories and the potential therapeutic role of the expressed genes have not been fully understood (8). There are, therefore, still substantial gaps in the clinical outcomes, encouraging scientists to seek further improvements in each of these issues. The differentially expressed genes (DEGs) associated with T2DM, such as *SRR*, *NFKB1*, and *PDE4B*, were examined in cases and controls to identify the genetic causes of the disease.

Based on quantitative PCR (qPCR)-validated genome- and transcriptome-level studies, *CHL1*, *LRFN2*, *RASGRP1*, *NFKB1*, and PPM1K have been substantially linked with insulin secretion and T2DM. In non-diabetic individuals, the influence of genetic diversity on continuous hyperglycemia events has largely shown that insulin secretion has been disturbed (9). Systems biology based on genome-wide association studies (GWAS) reveals the genetic reasons for T2DM pathogenesis and insulin resistance (9). Likewise, aberrant glucagon secretion resulted in inflammation of the islets in T2DM, and interleukin-6 has been found to stimulate secretion (10). Quantitative real-time PCR (qRT-PCR) is a method of choice in gene expression profiling and follow-up validation based on its high accuracy rate with an increased dynamic range and sensitivity (11, 12). An important technique to observe relative gene expression changes in qPCR studies is 2^-ΔΔC_T_^ . This approach is useful in assessing the relative levels of gene expression among various samples by using the qPCR technology directly to compute the threshold cycle (CT) (13).

Genome expression in insulin signaling and integrated pathology can alter any of these genes that might develop clinically important insulin resistance and diabetes (2). The systems biology method may combine these biological networks and assist in uncovering important etiological factors. As genes are crucial to a better understanding of the network of systems biology, complementary DNA (cDNA) microarray technology is an effective tool to simultaneously analyze the expression levels of thousands of genes. The significant number of expression datasets in the public field provides a valuable source of genome-wide information on T2DM and gives an opportunity to investigate the expression of a large number of samples. This work aimed to discover the insulin resistance and T2DM genetic variations by qPCR experimental validation. System-level analysis screened out the *SRR*, *NFKB1*, and *PDE4B* genes from cDNA datasets. qPCR analysis examined the dysregulation of these genes and their pathological role in T2DM. These findings would help in understanding the genetic basis of the disease and will update therapeutic strategies against T2DM.

## Materials and Methods

### Ethical Approval and Collection of Blood Samples

We collected a total of 100 individual blood samples with an equal ratio of control/cases (*n* = 50) from the local hospital in Multan, Punjab Province. For qPCR analysis, blood samples were collected from type 2 diabetic patients and healthy individuals based on inclusion and exclusion criteria. The approval of the study and informed consent were obtained from the Research Ethics Committee of the Institute of Molecular Biology and Biotechnology, Bahauddin Zakariya University, Multan (ref. no. IMBB/2019/002).

### Inclusion and Exclusion Criteria

We used 1) pathologically confirmed cases of T2DM; 2) new patients diagnosed by the Nishtar Hospital, Multan, for the first time; 3) patients aged ≥18 years; 4) patients who agreed to provide blood samples for scientific research and consent for the publication of research data; and 5) healthy individuals with no history of diabetes, cancer, and cardiovascular diseases, who were included as controls. The exclusion criteria were: 1) pathologically confirmed local vascular invasion; 2) cases with multiple and complex diseases; 3) cases with cancer or immune disorders; and 4) cases with a history of surgery in the past 3 years.

### Normalization and Differential Expression Analysis

Twenty T2DM-related cDNA expression datasets from the NCBI Gene Expression Omnibus (GEO) database were downloaded (**Supplementary Table S1**). DEGs were identified using the Affymetrix U133 Plus 2.0 Array platform and a hgu133plus2 annotation probe. The R platform with Affy, AffyQCReport, AnnotationDbi, Annotate, Biobase, Lima, and hgu133a2cdf Bioconductor packages were used to analyze the computable outcomes. AffyRNAdeg, summary AffyRNAdeg, and plotAffyRNAdeg were used for RNA degradation analysis and for checking the quality of RNA samples. Perfect match (PM) and mismatch (MM) were measured using the Robust Multi-Array Average (RMA) normalization approach, and normalization of the microarray datasets was utilized for comparison. These datasets were grouped into recognizable pheno-data files that include the accession numbers, sample types, number of samples, disease, and clinical conditions (14). RMA was applied for noise reduction from local signals (15, 16). RMA has been a frequently used method to generate an expression matrix from Affymetrix data. The raw expression values of genomic data were background corrected, log2 transformed, and then quantitatively standardized, followed by the linear model being applied to acquire an expression measure for each probe set on each cDNA array. Normalization was carried out to observe the perfect matches through the median level. For normalization and background correction, PM and MM, the following equation was applied:


$$\text{P}{\text{M}}_{ijk}=\text{ B}{\text{G}}_{ijk}+{\text{S}}_{ijk}$$

where PM indicates a perfect match, BG is “background,” *S* is nonspecific binding, and *ijk* represents the signal for probe *j* for the probe set *k* on microarray *i*.


$$\text{BG}(\text{P}{\text{M}}_{ijk})=E[S_{ijk}\;|\text{P}{\text{M}}_{ijk}]>0$$

$$S_{ijk}∼\text{Exp}(\lambda_{ijk})\text{ B}{\text{G}}_{ijk}∼N(\beta_{i},\sigma^{2})$$

In differential analysis, the PM highlights the communal signals of background (BG) and expression (*E*). The “ArrayQualityMetrics” Bioconductor package was used to measure the quality of the samples, indicating the median expression level. *N*(*β_i_*, *σ* ^2^) is the normal distribution involving *BG_ijk_*. In this case, *β_i_* is the mean regression parameter that assesses the independent variable gene expression *i*. *σ* ^2^ is the least squares regression representing the dependent variables based on the linear model. *λ_ijk_* is a distribution rate parameter that reads the transcription of lane *k* to gene *j* from sample *i*. The gene square matrix for each dataset was calculated across the microarrays and missing values were ignored. For a probe set, we utilized the RMA method to compute a summary of the values among samples.


$$X\text{norm}\;=F_{2}^{-1}(F_{1}(x))$$

where *F*_1_ and *F*_2_ show the distribution functions of the case and the reference DNA chips, respectively.

In this study, we detected the T2DM-related DEGs from each cDNA dataset by pairwise comparison (17). DEGs and duplicated spots with quality signals were shortlisted. The statistical parameters were recorded and the genes were ranked. False discovery rate (FDR) <0.05, *p*-value ≤0.05, average expression level (AEL) ≥40%, and an absolute log-fold change (LFC) >1 were used as significant cutoff values (18, 19).

### K-Fold Cross-Validation

We applied *k*-fold cross-validation to assess accuracy in the differential comparison using the Bioconductor “boot” package, and this method had the edges eventually using all samples for the training and test datasets (18). The *k*-fold approach is usually less biased in comparison with other methods because it ensures that all observations from the original dataset are shown in the training and test sets. In molecular studies, bootstrapping has been successfully used to correct mismatches and background noise (20). The generalized Gaussian linear model was used to check the *k*-fold cross-validation with the cv.glm technique. It calculates the true error as the average error.


$$E=1/k\sum_{i=1}^{k}Ei$$

The Gaussian rule is applied based on leave-one-out-cross-validation (LOOCV). In this case, the LOOCV procedure is considered the test set while the remaining data are used as a training set. LOOCV is a modified *k*-fold, where *k* = *N* and *k*(*i*) = *i*. For training and other testing, we applied *N* number of subsets. Increasing the number of samples would decrease errors and improve the validity (18). For the validation and assessment of errors, the following formula was used:


$$E=1/N\sum_{i=1}^{N}Ei$$

### Gene Ontology and Pathway Enrichment Analysis

Gene Ontology (GO) is a common categorization method used to define the key signaling pathways for the biological and molecular activities of DEGs and cellular components. To recognize the function and biological pathways of the T2DM-related DEGs, GO analysis was carried out using a GOnet online web server that shows the gene product and its biological functions (21). The pathway enriched terms (*p* < 0.05) were analyzed using the FunRich tool, version 3.1.3 (22).

### Identifying Transcription and Regulatory Motifs

To develop a connected transcription network consisting of transcription factors (TFs) and other signaling molecules, we anticipated the possible regulators of T2DM-related DEGs using the FunRich online tool. These gene regulators have different biological and pathological roles (23). The motifs are signatures of protein families that define the link between the secondary structural components of proteins, and, in every instance, the spatial sequences of the amino acid residues encoded by genes may be comparable in any order. We used the Motif Search online tool to analyze the relationship between the primary sequence and the tertiary structure of proteins to predict protein functions (24).

### Mutation Analysis

Mutations resulting from inherited and non-inherited diseases can be understood to decode genetic variations by the association of genotype with phenotype. There are hundreds of single nucleotide variations (SNVs) in the human genome, and many are known to develop diseases. Nearly 21% of the amino acid variations cause single nucleotide missense mutations at different protein sites (known as posttranslational modifications) that are responsible for disease development. Therefore, chemical alterations of amino acids mostly affect the functions of proteins. The online ActiveDriverDB tool was used to analyze the mutations of T2DM-related DEGs (25). In this analysis, the needle plot shows a visual summary of the location, frequency, and functionality of all alterations found in our DEGs. Posttranslational modification (PTM) sites with all changes and the predicted disordered regions of the protein sequences were analyzed. The position of the pins in the plot correlates with the gene and protein sequences, while the associated mutation effect and PTMs may be observed in the figure legend.

### Protein Co-Expression Network Analysis

Co-expression network analysis enables the identification of protein modules whose biological activities are characterized by expression patterns. These networks are the most versatile for probing diseases. It correlates at the transcript level, although it may also be used to analyze correlations at all biological levels. The STRING database version 11.0 (26, 27) was used to study the protein–protein interactions (PPIs) of the top-ranked T2DM-related DEGs, and a protein co-expression network was constructed to highlight the important gene signatures directly or indirectly interacting with the DEGs. The protein network based on neighborhood scoring was constructed with high confidence (score > 0.99) (26). In this co-expression network, the RNA levels and protein regulation were analyzed and annotated keywords (FDR < 0.05) were studied.

### RNA Extraction and Quantification

Each 300 μl blood sample was transferred to a 700-μl triazole-containing Eppendorf tube (1.5 ml). These tubes were homogenized and incubated gently at 25°C for 5 min. Then, 400 µl of chloroform was added and kept for 3 min, followed by centrifugation at 12,000 rpm for 10 min at 4°C for phase separation. Sequentially, the aqueous top layer was taken into a new tube while keeping it on ice with equal proportions of isopropyl alcohol. These tubes were retained on ice at −20°C for 10 min in a horizontal position to precipitate RNA, followed by centrifugation at 12,000 rpm (4°C) for 10 min while the supernatant was discarded. The pellet was washed twice and air dried for 5 min using 1 ml of 70% ethanol at 7,500 rpm. Forty microliters of RNase-free water was added and then RNA was stored at −80°C (28) using an RNA stabilizer. Finally, RNA was quantified at 260, 280, and 320 nm by Nanodrop (Skanit RE 4.1, Thermo Scientific, Waltham, MA, USA) (29).

### cDNA Synthesis

The isolated RNA was converted into cDNA using a cDNA synthesis kit (Vivantis cDSK01-050). As per the manufacturer’s protocol, each RNA primer of the DEGs was co-mixed with 10 µl of the cDNA synthesis mix. After centrifugation at 10,000 rpm, the samples were incubated at 40°C for 60 min. The tubes were then incubated at 85°C for 5 min to terminate the reaction. Finally, the tubes were chilled on ice and centrifuged at the same conditions. The synthesized cDNA was directly used for further analysis (29, 30).

### Quantitative Real-Time PCR Analysis

The 260:280 ratios between 1.5 and 2.7 indicated high-quality RNA with 800–1250 ng/μl quantity. RNA quantification presented a substantial level for further cDNA synthesis. The relative expressions of the T2DM-related genes *SRR*, *PDE4B*, and *NFKB1* were studied and estimated based on the relative $2^{{-\Delta \Delta \text{C}}_{\text{T}}}$ method. CT values were obtained by absolute quantification presenting quality and significant expression at the real level. We used PrimerBank, an online server, to design the primers of the T2DM-related DEGs (31) (**Table 1**). For optimization, gradient PCRs were carried out using a Galaxy-XP thermal cycler (Bioer, Hangzhou, China) at standard conditions. To validate the DEGs of T2DM, qRT-PCR was performed using MIC-PCR (Bio Molecular Systems, Upper Coomera, Australia) at optimized conditions (32). A final volume of 10 µl of the reaction mixture was prepared using 2.6 µl of cDNA (1:10), and 5 µl of the SYBR Green Master Mix and 0.4 µl of each gene-specific reverse and forward primer were added. The final composition of the reaction mixture was kept for DEGs and the reference genes. *GAPDH* was used as the internal reference, and a two-step qPCR procedure was applied to measure the expressions of the test and reference genes. The relative expression level of each differential gene was calculated with the *GAPDH* expression level as a “1” standard value. The qPCR cycling conditions were as follows: denaturation at 95°C for 12 min, 40 cycles of 95°C for 15 s, 57°C for 20 s, and 72°C for 20 s. A final extension step was carried out at 72°C for 10 min (33). In amplification, during each cycle, the amplified products were doubled in an exponential form. The PCR products were analyzed by melting the curve graph to assess the dissociation characteristics of double-stranded DNA during heating and to observe the absorbance intensity (34). *C*_T_ is a logarithmic value converted to a relative quantity (32), and the average *C*_T_ values were measured for both DEGs and the reference gene. In the next step, Δ*C*_T_ (delta threshold) was calculated for the target and the reference gene, and the relative expressions of the T2DM-related DEGs were studied and estimated based on the relative 2^−ΔΔC_T_^ formula.


$$\Delta C_{T}=C_{T}\text{value of target gene}-C_{T}\text{value of the internal reference gene (}GAPDH)$$

**Table 1:** Primer sequences and amplicon size of the differentially expressed genes *SRR*, *NFKB1*, and *PDE4B* used in the qPCR reaction.

Gene symbol	Probe ID	Protein name	Forward primer	Reverse primer	Amplicon size (bp)
*SRR*	219204_s_at	Serine racemase	ATGTGTGCTCAGTATTGCATCTC	AAGATTGCGCCCTGTTAGTTG	126
*NFKB1*	201502_s_at	Nuclear factor NF-kappa-B p105	AACAGAGAGGATTTCGTTTCCG	TTTGACCTGAGGGTAAGACTTCT	104
*PDE4B*	203708_at	cAMP-3′,5′-cyclic phosphodiesterase 4B	AACGCTGGAGGAATTAGACTGG	GCTCCCGGTTCAGCATTCT	110
*GAPDH* (reference gene)	–	Glyceraldehyde-3-phosphate dehydrogenase	GGAGCGAGATCCCTCCAAAAT	GGCTGTTGTCATACTTCTCATGG	197

The ΔΔ*C*_T_ (delta–delta threshold) specifies the differences between the expression levels of the T2DM-related DEGs and the reference gene (35). Finally, $2^{{-\Delta \Delta \text{C}}_{\text{T}}}$ was calculated, indicating the fold difference of the expressions of DEGs from that of the control (36). We assessed the absolute correlation of these DEGs to show the complete expression levels. Hierarchical clustering analysis of genes to illustrate the expression pattern was observed (37) using the online one-matrix CIMminer tool (38).

## Results

### Normalization of Gene Expression Data and Cross-Validation

**Figure 1:**
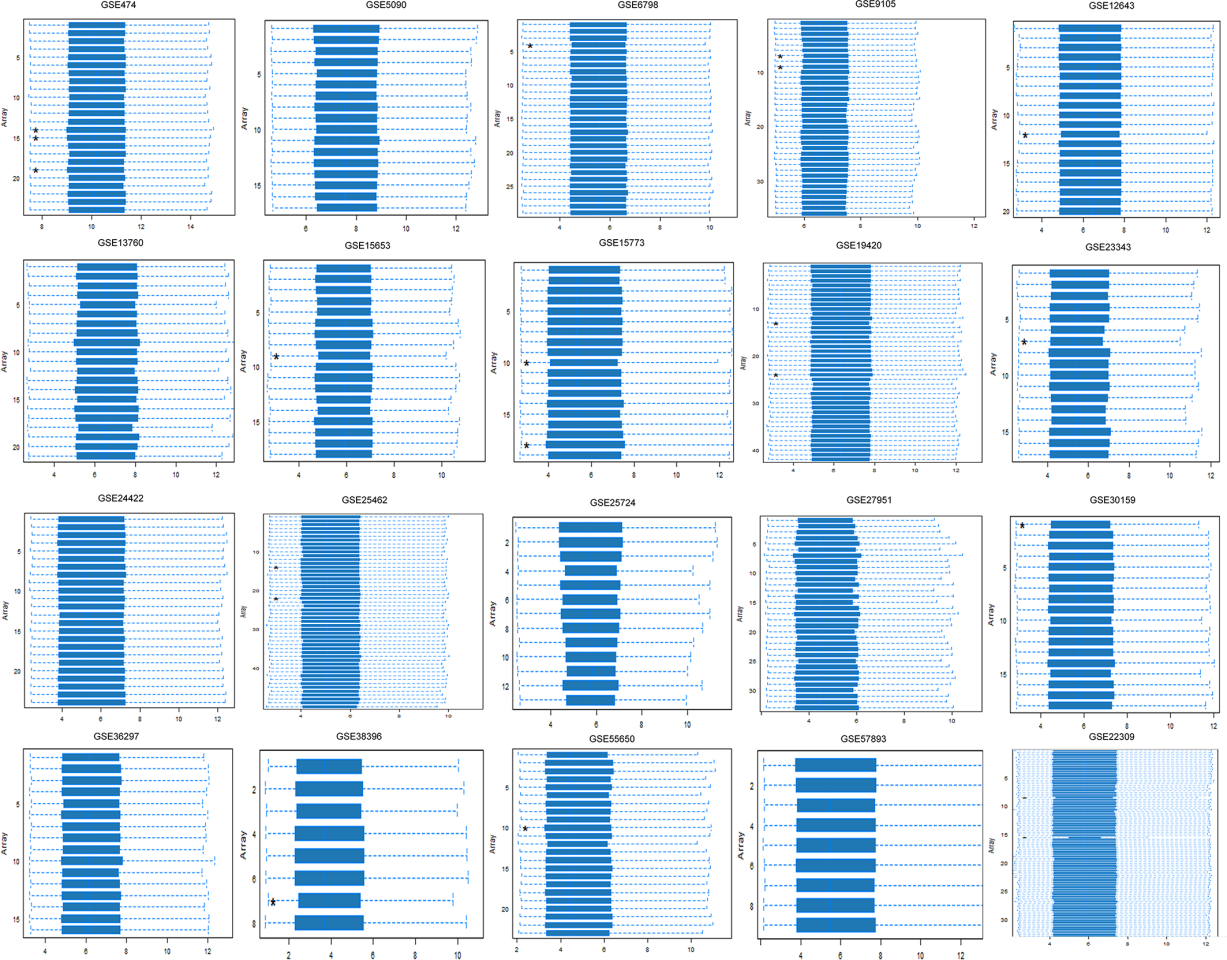
Array intensity distributions showing boxplots representing summaries of the signal intensity distributions of the arrays and normalization with similar positions and widths. Each *box* corresponds to one array. Outlier detection was performed by computing the Kolmogorov–Smirnov *K*_a_ statistic between each distribution of the array and the distribution of the pooled data.

**Figure 2:**
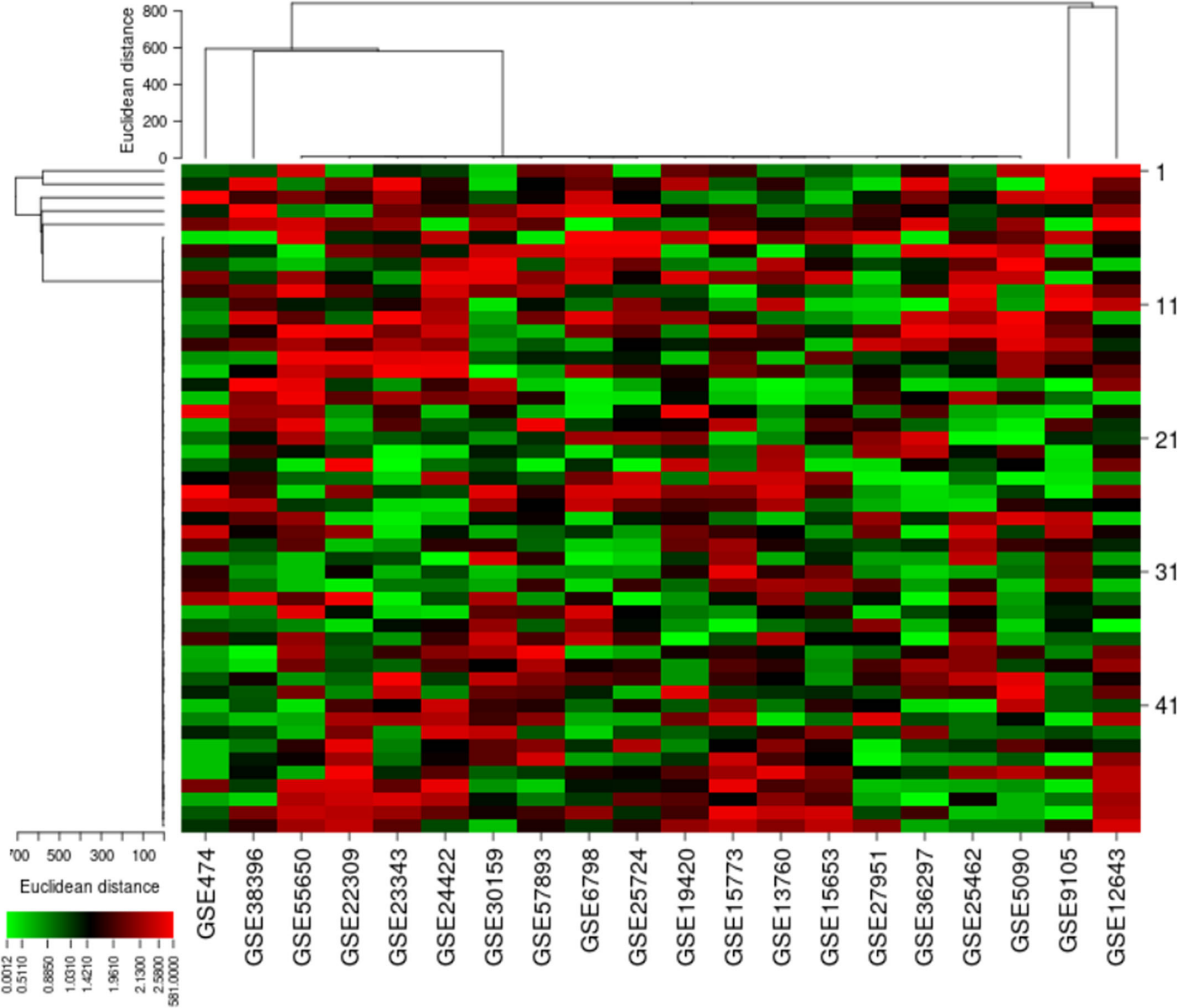
Differential expressions of 50 genes obtained from microarray cDNA datasets. The heat map indicates the differential expressions of these genes in each type 2 diabetes mellitus (T2DM)-related dataset.

To study the T2DM-related DEGs, we retrieved 20 publicly available Affymetrix cDNA datasets. Each dataset contains a number of distinct samples and genes obtained by messenger RNA (mRNA) expression profiling utilizing several Affymetrix T2DM platforms. The data were normalized missing values were corrected, and the normalized distance between the DNA chip array and the different arrays of each dataset reveals the quality of the arrays for a medium expression level. The genegene covariance matrix in all arrays of every dataset was calculated by removing the missing values to determine whether the arrays were on the same level. A quality histogram depicting the normalized intensity arrays of the entire DNA chip was constructed during the quantile standardization. Array intensity distributions show boxplots representing summaries of the signal intensity distributions of the arrays and normalization with similar positions and widths. The patterns revealed the distribution of the arrays having similar shapes and ranges (**Figure 1**). From the list of 50 DEGs (**Figure 2**), we identified and selected three DEGs of T2DM—*SRR*, *NFKB1*, and *PDE4B*—based on the FDR (<0.05), *p*-value (≤0.05), and logFC (>1) parameters.

An automated method was employed for comparing biologically similar groups in pairs. We excluded any subgroup without recurrence comparison, accuracy, and differential analysis verification and assessed errors of the cross-validation using the generalized linear model “cv.glm.” This generalized linear model normalizes the linear regression, enabling the linear model to be associated with the dependent variables through a correlation and allowing a function of the anticipated value of the variance of each measurement. The Gaussian dispersion criterion was 0.00459, indicating the degree of confidence (**Table 2**). LOOCV is a type of cross-validation technique where each observation is treated as a validation set and the remaining observations (*N* − 1) as a training set. The model was fitted in LOOCV and a validation set was used to estimate it. With *k*-fold valuation, we found the same delta value of 0.0050 we used in the LOOCV approach. The significant codes (0.1, 0.01, 0.001, and 0.05) with residuals of low variance showed that the differential analysis was consistent. We evaluated the confidence of the dataset in the original samples in order to identify the variations at the transcription level. The normalization procedure was utilized to standardize the sampling methods and to evaluate the optimum RNA quality by utilizing refined measures to analyze statistics and algorithms.

**Table 2:** *K*-fold cross-validation using the Bioconductor “boot” package based on Gaussian dispersion parameters.

	Estimate	SE	*t* value	Pr(>|*t*|)
(Intercept)	0.000107	0.000211	3.99	<1.00E^−13^***
*x*_1_	0.030034	0.001602	20.018	<1.00E^−11^***
*x*_2_	−0.01041	0.001105	−4.017	<1.96E^−10^***
*x*_3_	0.110112	0.003101	22.015	<1.00E^−13^***
*x*_4_	0.110410	0.001212	20.200	<1.00E^−11^***
*x*_5_	0.016013	0.002130	28.003	<1.00E^−10^***
*x*_6_	0.131220	0.003361	21.012	<1.00E^−10^***
*x*_7_	−0.01201	0.001461	−21.112	<1.00E^−9^***
*x*_8_	0.001212	0.002411	19.115	<1.00E^−12^***
*x*_9_	0.102111	0.003802	62.0716	<1.00E^−13^***
*x*_10_	0.010020	0.000500	4.001	<1.00E^−11^***
*x*_11_	0.010521	0.001003	22.003	<1.00E^−11^***
*x*_12_	−0.01102	0.002014	−2.014	0.0068*
*x*_13_	−0.12421	0.002809	−50.023	<1.00E^−7^***
*x*_14_	0.010021	0.001230	1.312	5.28E^−8^***
*x*_15_	−0.015581	0.001200	−17.102	<1.00E^−11^***

Number of Fisher scoring iterations: 2; $K: [1] 10; $delta: [1] 0.00455 = 0.00459. Null deviance: 100,502.1 with 50,101 degrees of freedom. Residual deviance: 2,504.1 with 40,119 degrees of freedom.

Signif. codes: 0.001 ‘**’.

### Gene Ontology and Pathway Enrichment Analysis

**Figure 3:**
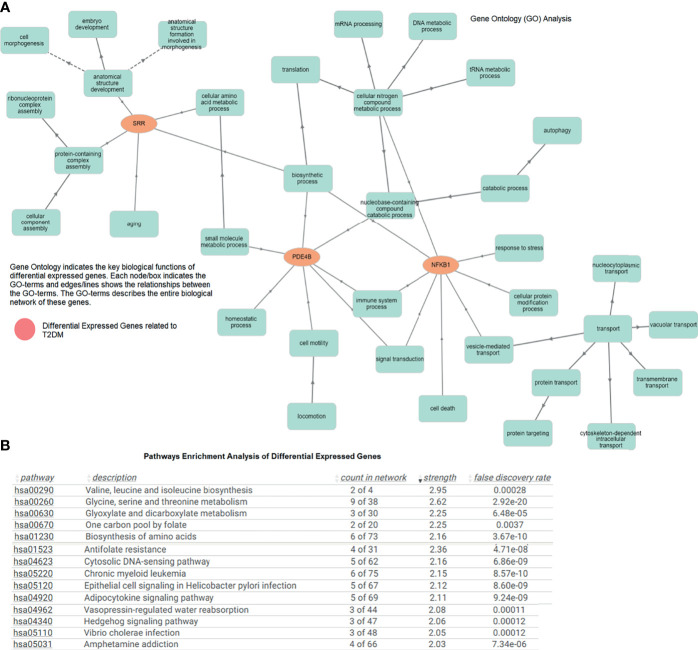
**(A)** Gene Ontology network of the differentially expressed genes indicating important biological functions. **(B)** Pathway enrichment analysis showing the enriched terms associated with type 2 diabetes mellitus (T2DM).

The GO of the T2DM-related DEGs showed significantly enriched terms. *SRR*, *PDE4B*, and *NFKB1* were directly and indirectly linked to cell morphogenesis, anatomical structure development, amino acid metabolism, biosynthetic and catabolic processes, mRNA processing, signal transduction, protein transport, transmembrane and intracellular transport, and other vital biological functions (*p* < 0.05). The molecular annotation of *SRR* showed associations with ATP, magnesium, phosphate, calcium, and serine–ammonia lyase binding, and threonine racemase activity. Similarly, at the molecular level, *NFKB1* and *PDE4B* were involved in actinin chromatin binding, transcription regulation, and cAMP-binding phosphodiesterase activity. The role of the gene, its regulation, subtypes, and the cellular processes are essential in the comprehension of biological functions, and abnormalities in these processes can cause T2DM and other metabolic disturbances (**Figure 3A**). The pathway enrichment showed the role of valine, leucine, and isoleucine biosynthesis; glycine, serine, and threonine metabolism; the adipocytokine signaling pathway; the Hedgehog signaling pathway; and insulin and antifolate resistance in T2DM (**Figure 3B**). These DEGs associated with signaling pathways are critically linked to pathophysiological mechanisms.

### Transcription and Motif Analysis

**Figure 4:**
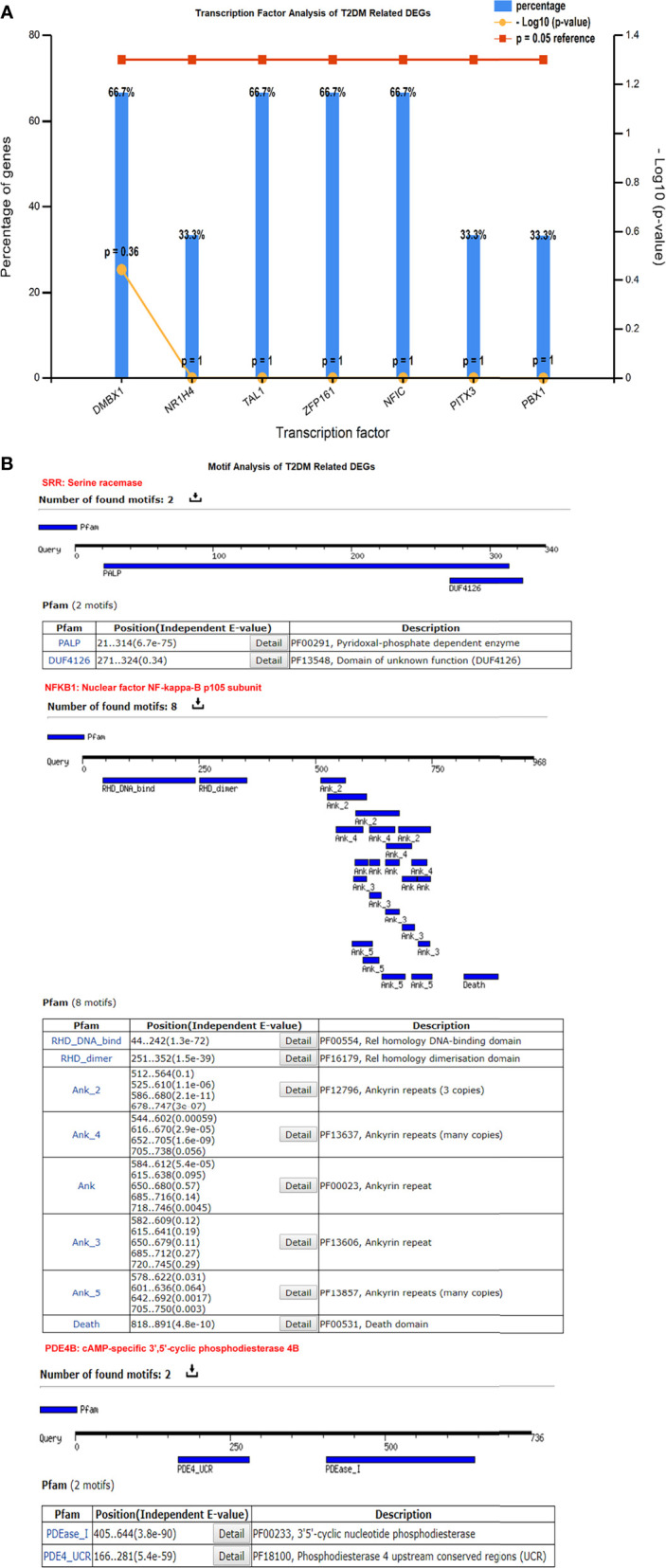
**(A)** Transcriptional factors of the differentially expressed genes (DEGs) showing *DMBX1*, *TAL1*, *ZFP161*, *NFIC*, and other regulatory factors. **(B)** Motif analysis highlights a significant number of functional motifs associated with important biological functions.

We identified the transcriptional factors of the T2DM-related DEGs, such as *DMBX1*, *TAL1*, *ZFP161*, *NFIC* (66.7%), and *NR1H4* (33.3%), and others with a substantial *p*-value (<0.05) (**Figure 4A**). These motifs play a crucial role in the protein interactions among network components. These motifs help to discover TF binding sites and expression of gene regulation. The outcomes showed that the remodeling of a variety of motifs established the promiscuous protein characteristic resulting in different biological functions. We found eight motifs of the nuclear factor NF-kappa-B subunit (NFKB1) protein containing Pfam-annotated DNA binding, cell death, dimerization domain, and ankyrin repeats (proteins that facilitate the attachment of fundamental membrane proteins). Pfam annotation was predicted based on multiple sequence alignments and a hidden Markov model. Under diabetic conditions, the aberrant expression of *NFKB1* ankyrin repeats dysregulate glucose homeostasis and protein kinase (AMP) activity. For the other DEGs, *SRR* contained PALP and DUF4126, while *PDE4B* had the PDEase_1 and PDE4_UCR motifs. These motifs are biologically associated with cyclic nucleotide phosphodiesterase and pyridoxal phosphate-dependent enzyme activity. The dysregulation of these transcriptional regulators changes the phosphodiesterase activity, resulting in insulin resistance and T2DM. The motifs were scanned with a significant cutoff value of *e* < 0.0002 at the default parameters (**Figure 4B**).

### Mutation Analysis

**Figure 5:**
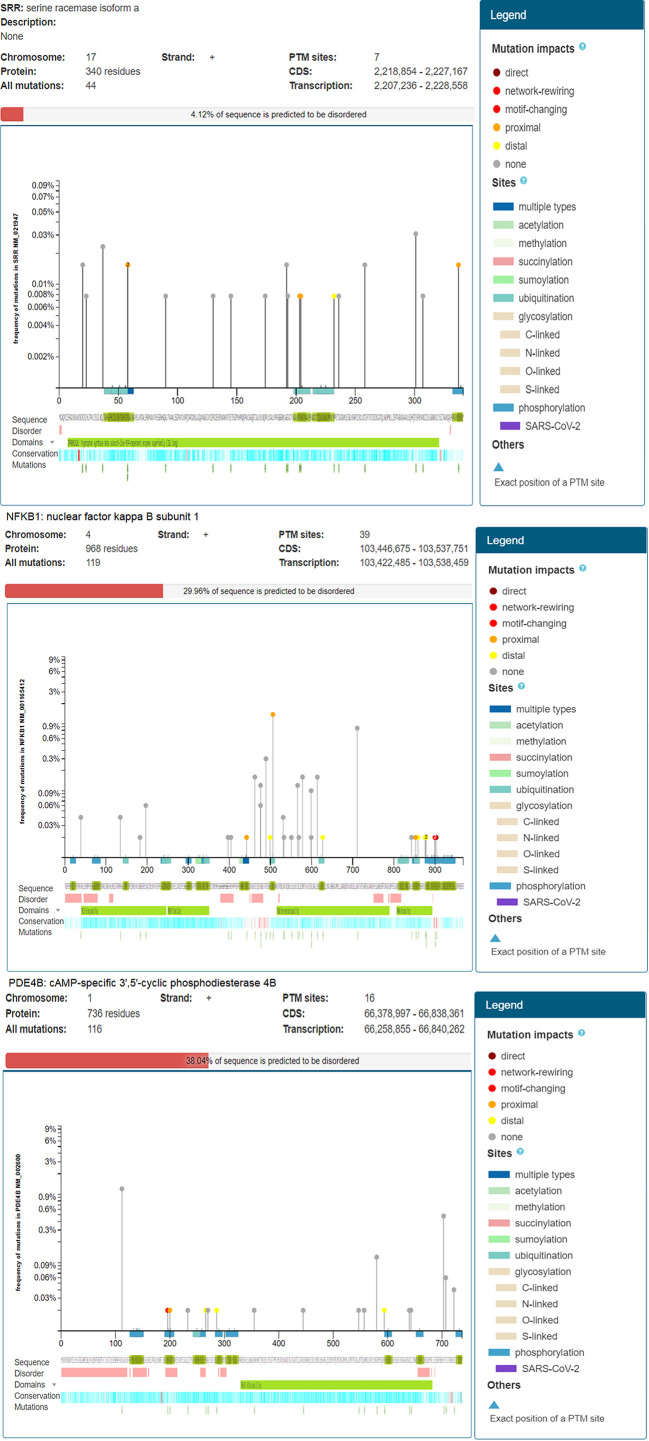
Mutation analysis of type 2 diabetes mellitus (T2DM)-related differentially expressed genes (DEGs) indicating posttranslational modifications with significant cutoff parameters. It highlights the significant disordered regions of the proteins contributing a pathophysiological role and to disease development.

*NFKB1* had 39 PTM sites with 119 repeated mutations at the positive strand of chromosome 4 encoding 968 protein residues, indicating 29.96% of the predicted disordered region. The mutation visualization plot displayed *NFKB1* isoforms distal, proximal, direct, and network-rewiring mutation impacts between 450 and 900, with reference amino acid (AA) residues Q (Gln), D (Asp), N (Asn), M (Met), H (His), and A (Ala) and mutated amino acid residues E (Glu), K (Lys), R (Arg), D (Asp), T (Thr), and Y (Tyr), and others in the protein. The mutated site of NFKB1-Q900E at position 897 contained S amino acid residues enriched with a phosphorylation network-rewiring mutation impact. *NFKB1* D442Y contained K residues with acetylation and ubiquitination proximal mutation impacts at the 440 position of a protein. On the other hand, *PDE4B* showed 38% disordered regions in a sequence. The *PDE4B* R196S isoform revealed the mutation at position 197 presenting amino acid residue R to the mutated amino acid residue S. At this position, the S amino acid residue sites enriched with phosphorylation indicated network-rewiring PTM impact. PDE4B-A200V contained the S residue with a phosphorylation proximal mutation impact at the 201 position of a protein. Similarly, the differentially expressed *SRR* gene showed a 4.12% disordered region with 44 mutations predicted at chromosome 17 on the positive strand involving 340 amino acid residues and seven PTM sites. The *SRR* R58G isoform showed the mutation at position 58 presenting amino acid residue R to the mutated amino acid residue G with ubiquitination, indicating proximal PTM impact (**Figure 5**). Similarly, at position 203 of *SRR* proteins, the AA residue S was altered by N, indicating proximal PTM impact.

### Protein Product Co-Expression Network Analysis

**Figure 6:**
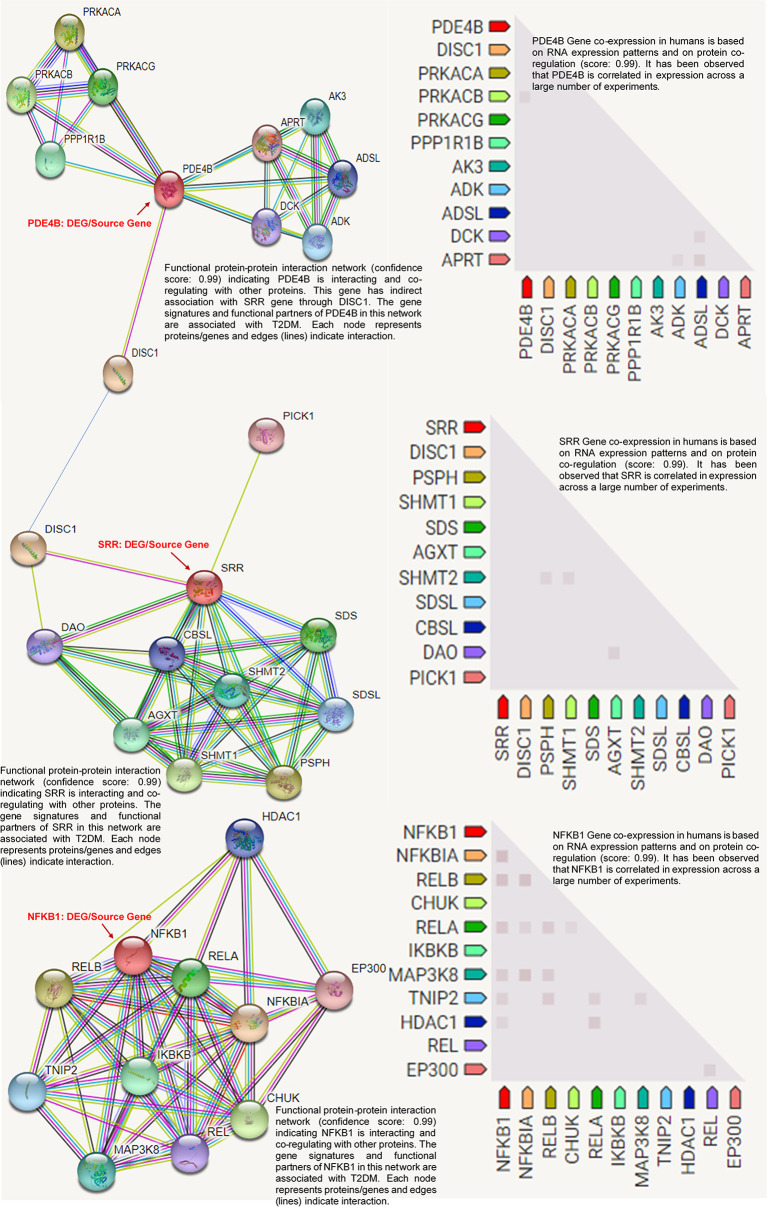
Protein product co-expression network analysis using the STRING database, version 11.0. The protein network was calculated based on the neighborhood score with higher confidence (confidence score > 0.99). *Nodes* represent proteins and *edges* indicate interactions. The co-expression scores based on RNA expression patterns and protein co-regulation were studied and annotated keywords (FDR < 0.05) were observed.

The *SRR*, *PDE4B*, and *NFKB1* genes were studied for possible interactions with each other using the STRING database. It was predicted that these DEGs would have significant interactions. The PPI network contained 33 numbers of nodes (each node indicates proteins), and the edges present the interactions. The *PDE4B* network showed the enriched co-expressed genes (PPI enrichment, *p* < 0.05) functionally associated with adenine phosphoribosyl transferase (*APRT*), deoxycytidine kinase (*DCK*), adenyl succinate lyase (*ADSL*), AMP phosphotransferase (*AK3*), protein phosphatase-1 regulatory subunit 1B (*PPP1R1B*), adenosine kinase (*ADK*), and cAMP-dependent protein kinase catalytic subunit alpha (*PRKACA*). *PDE4B* is directly connected to the *SRR* source gene *via* DISC1 (schizophrenia 1 protein). *SRR* is linked to d-amino-acid oxidase (*DAO*), l-serine ammonia-lyase (*SDS*), cystathionine beta-synthase-like protein (*CBSL*), phosphoserine phosphatase (*PSPH*), and other gene signatures. Based on the protein product co-expression data, we evaluated the expressions of the genes *SRR*, *PDE4B*, and *NFKB1A* and observed that these were downregulated. Similarly, it has been observed that *NFKB1A* interacts with important target proteins such as transcription factor A (RELA), TNFAIP3-interacting protein 2 (*TNIP2*), an inhibitor of nuclear factor kappa-B kinase subunit alpha (*CHUK*), an inhibitor of nuclear factor kappa B kinase subunit beta (*IKBKB*), and other gene signatures. The RNA co-expression pattern and protein co-regulation showed the significant level of association of the co-expressed genes with annotated keywords: hyperlipidemia, very low-density lipoprotein (VLDL), low-density lipoprotein (LDL), high-density lipoproteins (HDL), lipid metabolism, and disease mutation (**Figure 6**).

### Validation qRT-PCR Assay and Expression Profiling

**Figure 7:**
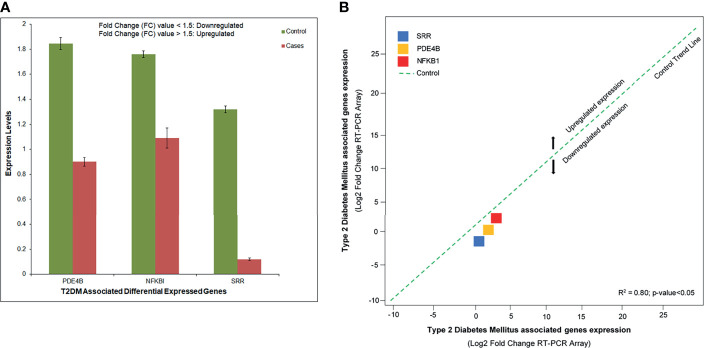
**(A)** Abnormal expression levels of the differentially expressed genes (DEGs) in type 2 diabetes mellitus (T2DM) cases and controls based on the fold change of gene expression. **(B)** Quantitative real-time PCR (qRT-PCR) array validation of the DEGs. Plot graph shows the correlation between the expression levels of type 2 diabetes mellitus (T2DM)-related DEGs measured by array analysis and the expression levels measured by individual qRT-PCR. The 2^−ΔΔC_T_^ method was applied for this analysis.

The RT-PCR was consistent enough to prevent the reaction from displaying an increased fluorescence signal. The relative expression showed that *SRR*, *NFKB1A*, and *PDE4B* were downregulated with significant fold change values $\left(2^{{-\Delta \Delta \text{C}}_{\text{T}}}\right)$ compared to controls. These DEGs were aberrantly expressed and related to disease development and growth (**Figure 7A**). Based on the 2^−ΔΔC_T_^ method, we observed a substantial level (*R* ^2^= 0.80, *p* < 0.05) of the relationship between the expression levels of the T2DM-related DEGs assessed by array analysis and the expression levels evaluated by individual qRT-PCR (**Figure 7B**).

**Figure 8:**
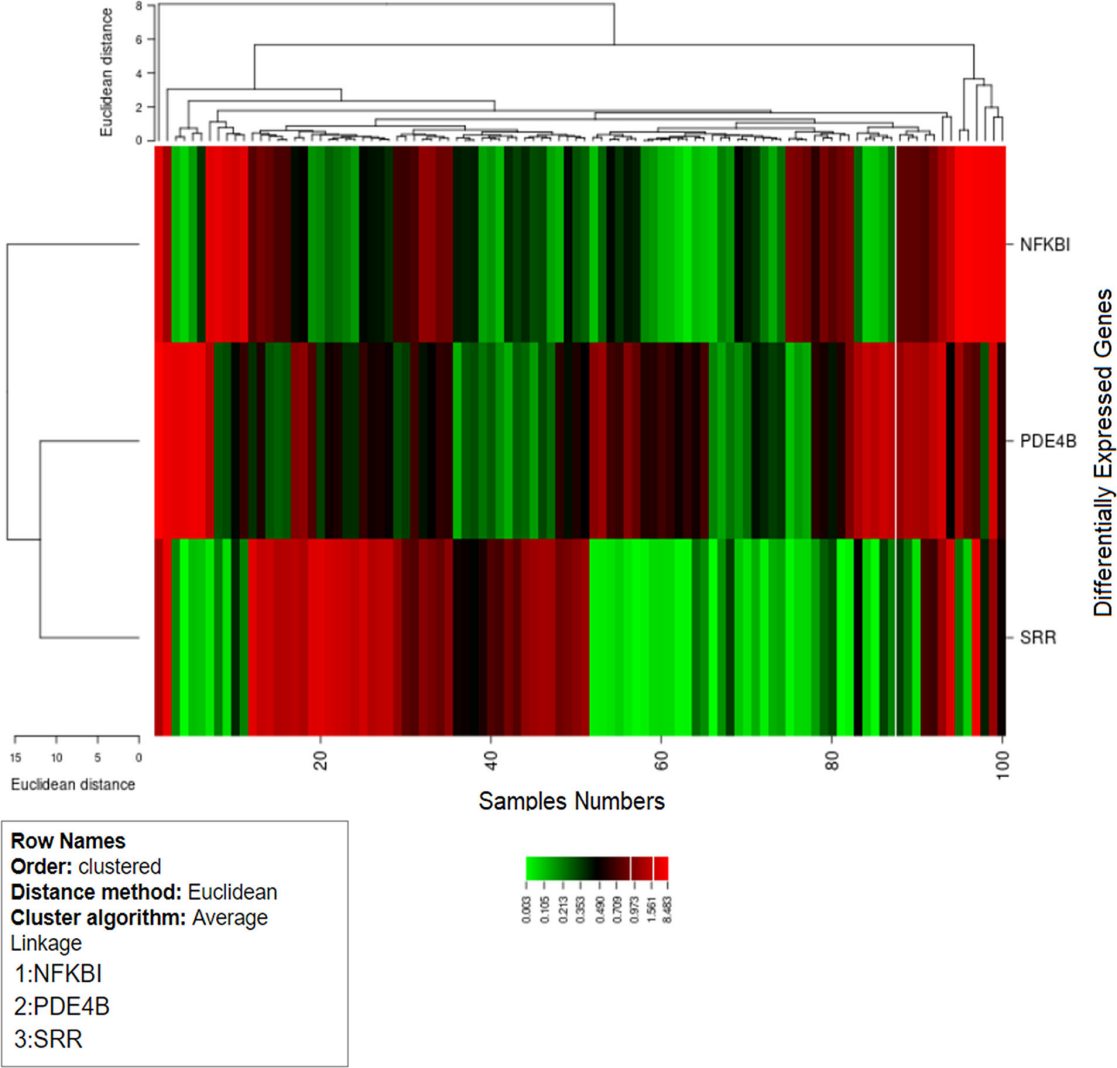
Hierarchical cluster analysis heat map indicates the expression profiles of differentially expressed genes (*SRR*, *NFKB1*, and *PDE4B*). The *columns* in the figure represent the sample numbers and the rows represent the differentially expressed genes.

In our analysis, the cluster study specified the gene expression profiles of the cases and controls (two groups) with significant expression level differences. **Figure 8** shows that most of the samples have differential expression profiles compared to the left-hand dendrogram, where some genes have similar expression patterns (**Figure 8**). The genes *NFKB1A*, *SRR*, and *PDE4B* were analyzed in 50 cases and controls, and the expression levels of the cases and controls (log fold values) were studied individually and indicated on a heat map. In this figure, the columns indicate the samples and the rows represent the DEGs. These genes may have a parallel biological function or contribute to the same physiological role. Genes with increased differential expression were termed as a gene cluster, and we found important gene clusters that were expressed differently in a number of samples (fold change ≥2 and *p* < 0.05).

## Discussion

In developing countries, T2DM risk factors are frequently recorded, and the prevalence of diabetes is increasing, yet its control worldwide is insufficient (39, 40). Human gene expression has been shown to be of significance for the identification of phenotypic genetic determinants and for discovering complex genetic features. Many genes interact with the most prevalent conditions in individuals and thus need an integrative biological approach to resolve the complications and causes behind these issues (41). Progress in microarray analysis enabled scientists to simultaneously examine a large number of genes and to uncover genetic evidence for different diseases (42). In this study, publicly accessible cDNA datasets were analyzed to identify the DEGs for T2DM. It was revealed that *SRR*, *PDE4B*, and *NFKB1* variants were involved in interactions with known T2DM-associated genes, including *AK3*, *DISC1*, *ADK*, *ASPH*, *SDS*, *RELB*, *HDAC1*, *REL*, and *EP300*. The dysregulation and functional abnormality of these differential genes have been investigated in T2DM development (43–45).

The ontology of these genes and pathway analysis have demonstrated insulin resistance, insulin and adipokine signaling, amino acid metabolism, and T2DM. The amino acid metabolism, biosynthetic and catabolic processes, signal transduction, and other vital molecular functions were directly and indirectly associated with *SRR*, *PDE4B*, and *NFKB1*. We observed that PIk3, S1P1, endothelin, CALM1, TGFB, IL, STAT6, and the adipocytokine signaling pathways linked to these DEGs (40, 44, 46) were regulated by transcriptional factors such as *DMBX1*, *TAL1*, *ZFP161*, *NFIC*, and *NR1H4*. The co-expression network showed the direct and indirect functional interactions of the gene signatures with the source genes.

The diverse role of the family of *NFKB1A* genes has been studied in type 2 diabetes (44) associated with impairment of glucose metabolism. *NFKB1A* is the gene expression regulatory factor for many pro-inflammatory proteins. Animal studies have shown that the dysregulated and increased activity of this gene causes the pathogenesis of insulin resistance and muscle atrophy (47). It has been observed that amplified *NFKB1A* signaling may be involved in the pathogenesis of insulin resistance. The modified activity of this TF is connected to muscle loss and weakness (48), common characteristics observed in diabetic individuals.

Increased expressions of interleukin (IL)-1β and *NFKB1* and enhanced infiltration of macrophages have been observed in pancreatic islets of patients with T2DM. Methylation of the *NFKB1* gene was negatively correlated with the levels of IL-1Ra in individuals with T2DM (49).

cDNA differential expression analysis and integrative enrichment studies indicated the substantial association of IL-6, *NFKB1*, and *PIK3CG* with T2DM (50). *NFKB1*, *USF2*, *HINFP*, *MEF2A*, and *SRF* are important genes that are differentially expressed in T2DM (51). In another study, it has been observed that *NFKB1* is engaged in the generation of mild inflammation and oxidative stress, which cause diabetic issues (52).

Similarly, we observed the association of the *PDE4B* gene with cyclic AMP and the *SRC*, *INSRR*, *GRIN*, *DISC1*, *PRKACA*, *PRKACB*, *AK3*, *ADK*, and *APRT* gene signatures, their dysregulation causing insulin resistance and type 2 diabetes (53–55). PDEs are a large family of phosphodiesterases that catalyze cAMP and cGMP to 5′-AMP and 5′-GMP, respectively. It plays a vital role in intracellular signaling pathways and is linked to the regulation of glucagon-like peptide-1 release (56). The dysregulation of this enzyme leads to T2DM (57), making it a potential therapeutic target. PDE4 controls the level of cAMP and forms cAMP signaling, which delicately directs signals from various environmental stimuli to specific microenvironments. PDE4-cAMP signaling dysregulation signifies an important pathophysiological path in a metabolic disorder, as shown by its important role in processes such as inflammation, lipid and glucose metabolism, hepatic steatosis, abnormal lipolysis, and disturbed neuroendocrine functions (58). It has been observed that PDE3A, PDE3B, PDE4B, PDE4D, and PDE8B in rat islets and in INS-1E cells activate multiple signal pathways critical for pancreatic beta cell function, and their abnormalities are associated with insulin resistance (53). It has been reported that *PDE4B* is a potential therapeutic target to treat obesity-related metabolic diseases. These findings from rodent studies showed that genetic and pharmacological variations in *PDE4B* lead to insulin resistance, insulin and adipocytokine secretion, and T2DM (59).

**Figure 9:**
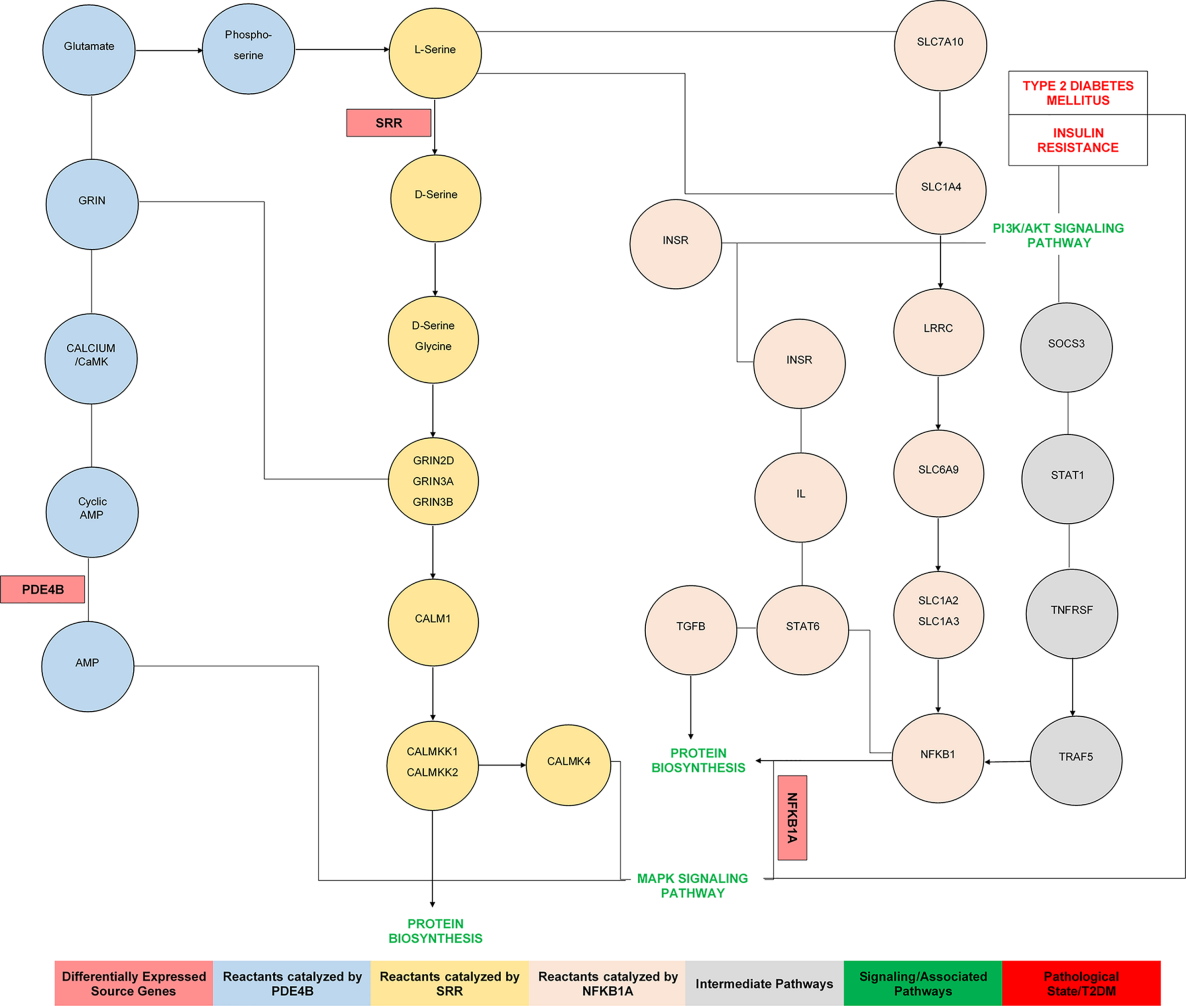
The pathways analysis indicates the mechanism and functional association of source genes involved in the pathophysiology of insulin resistance and type 2 diabetes mellitus.

In another study, it has been observed that the serine racemase (*SRR*) gene is involved in type 2 diabetes (60). GWAS have highlighted the polymorphic association of the *SRR* gene with T2DM (61). This analysis has identified >100 loci independently contributing to T2DM risk, signifying the evidence for a role in insulin secretion of T2DM susceptibility genes, including *PRC1*, *GRIN*, *SRR*, *ZFAND3*, and *ZFAND6* (62). Collectively, we found that the genes *SRR*, *PDE4B*, and *NFKB1A* were directly and indirectly associated with insulin secretion, insulin resistance, and T2DM (49, 58, 63). The pathway analysis diagram (**Figure 9**) showed that source DEGs were primarily enriched in cAMP signaling, amino acid metabolism, protein biosynthesis, the MAPK signaling pathway, regulation of lipolysis in adipocytes, the PI3K/Akt signaling pathway, insulin resistance, and T2DM (40, 57, 62, 64, 65). qPCR analysis was used to validate the outcomes of the differential analysis indicating the significant relationship of *SRR*, *PDE4B*, and *NFKB1* with T2DM. A significant fold variation was observed in the target samples using the $2^{{-\Delta \Delta \text{C}}_{\text{T}}}$ method (66), and real-time expressions of the T2DM-related DEGs demonstrated substantial level expression compared to control.

## Conclusions

In this study, we found a significant correlation of the DEGs *SRR*, *PDE4B*, and *NFKB1* with T2DM in cases compared to controls. Systems biology analysis of the cDNA data allowed us to discover potential therapeutic targets for T2DM, and our comprehensive and integrated steps were helpful in revealing the genome to phenome association with diabetes. The qPCR-based validation and expression profiling of these genes specified their abnormal expression and relates their pathological role in the disease process. These findings clearly demonstrate that the dysregulated expressions of selected genes are correlated with the pathophysiology of T2DM. Therefore, these genes would be considered as possible drug targets that would help update the therapeutic strategies for insulin resistance, T2DM, and other metabolic disorders.

## Data Availability Statement

The datasets presented in this study can be found in online repositories. The names of the repository/repositories and accession number(s) can be found in the article/**Supplementary Material**.

## Ethics Statement

The approval of the study and informed consent were obtained from the Research Ethical Committee of Institute of Molecular Biology and Biotechnology, BZ University, Multan (ref. no. IMBB/2019/002). The patients/participants provided written informed consent to participate in this study.

## Author Contributions

WR and JG collected the materials and performed the work. MQ and SM designed the study, interpreted the data, and wrote the manuscript. MQ and SM supervised the study. BB interpreted the data. All authors contributed to the article and approved the submitted version.

## Funding

This study was funded and supported by the Higher Education Commission (HEC) of Pakistan under Award Project no. 6913/Punjab/NRPU/R&D/HEC/2017. This funding body had no role in the design of the study and collection, analysis, and interpretation of data and in writing the manuscript.

## Conflict of Interest

The authors declare that the research was conducted in the absence of any commercial or financial relationships that could be construed as a potential conflict of interest.

## Publisher’s Note

All claims expressed in this article are solely those of the authors and do not necessarily represent those of their affiliated organizations, or those of the publisher, the editors and the reviewers. Any product that may be evaluated in this article, or claim that may be made by its manufacturer, is not guaranteed or endorsed by the publisher.

## Supplementary Material

Supplementary Table 1 List of cDNA datasets analyzed in this study.
