# Supplementary material for: qPCR Analysis Reveals Association of Differential Expression of SRR, NFKB1, and PDE4B Genes With Type 2 Diabetes Mellitus
Source: Front Endocrinol (Lausanne). 2022 Jan 3;12:774696. doi: 10.3389/fendo.2021.774696 (PMC8761634; doi:10.3389/fendo.2021.774696)
Supplement: Supplementary Table 1 — List of cDNA datasets analyzed in this study. [file Table_1.docx]

**SUPPLEMENTARY TABLE 1**. List of cDNA datasets analyzed in this study.

| S. No. | Accession Numbers | Samples  (Cases: Controls) | Web Link |
| --- | --- | --- | --- |
| 1 | GSE6798 | 16:13 | https://www.ncbi.nlm.nih.gov/geo/query/acc.cgi?acc=GSE6798 |
| 2 | GSE9105 | 36 in 3 conditions | https://www.ncbi.nlm.nih.gov/geo/query/acc.cgi?acc=GSE9105 |
| 3 | GSE12643 | 10:10 | https://www.ncbi.nlm.nih.gov/geo/query/acc.cgi?acc=GSE12643 |
| 4 | GSE13760 | 10:11 | https://www.ncbi.nlm.nih.gov/geo/query/acc.cgi?acc=GSE13760 |
| 5 | GSE15653 | 9:9 | https://www.ncbi.nlm.nih.gov/geo/query/acc.cgi?acc=GSE15653 |
| 6 | GSE15773 | 9:9 | https://www.ncbi.nlm.nih.gov/geo/query/acc.cgi?acc=GSE15773 |
| 7 | GSE22309 | 110 in 3 conditions | https://www.ncbi.nlm.nih.gov/geo/query/acc.cgi?acc=GSE22309 |
| 8 | GSE23343 | 10:07 | https://www.ncbi.nlm.nih.gov/geo/query/acc.cgi?acc=GSE23343 |
| 9 | GSE24422 | 12:12 | https://www.ncbi.nlm.nih.gov/geo/query/acc.cgi?acc=GSE24422 |
| 10 | GSE25462 | 25:25 | https://www.ncbi.nlm.nih.gov/geo/query/acc.cgi?acc=GSE25462 |
| 11 | GSE25724 | 6:07 | https://www.ncbi.nlm.nih.gov/geo/query/acc.cgi?acc=GSE25724 |
| 12 | GSE27951 | 45 | https://www.ncbi.nlm.nih.gov/geo/query/acc.cgi?acc=GSE27951 |
| 13 | GSE30159 | 9:09 | https://www.ncbi.nlm.nih.gov/geo/query/acc.cgi?acc=GSE30159 |
| 14 | GSE36297 | 6:10 | https://www.ncbi.nlm.nih.gov/geo/query/acc.cgi?acc=GSE36297 |
| 15 | GSE38396 | 04:04 | https://www.ncbi.nlm.nih.gov/geo/query/acc.cgi?acc=GSE38396 |
| 16 | GSE55650 | 12:11 | https://www.ncbi.nlm.nih.gov/geo/query/acc.cgi?acc=GSE55650 |
| 17 | GSE57893 | 9 | https://www.ncbi.nlm.nih.gov/geo/query/acc.cgi?acc=GSE57893 |
| 18 | GSE474 | 8 in 3  conditions | https://www.ncbi.nlm.nih.gov/geo/query/acc.cgi?acc=GSE474 |
| 19 | GSE19420 | 42 | https://www.ncbi.nlm.nih.gov/geo/query/acc.cgi?acc=GSE19420 |
| 20 | GSE5090 | 09:08 | https://www.ncbi.nlm.nih.gov/geo/query/acc.cgi?acc=GSE5090 |
